# Short-Term Efficacy of Using a Novel Low-Volume Bone Marrow Aspiration Technique to Treat Knee Osteoarthritis: A Retrospective Cohort Study

**DOI:** 10.1155/2022/5394441

**Published:** 2022-11-15

**Authors:** Daniel Kuebler, Alexandra Schnee, Lisa Moore, Jason Kouri, Alexis McLaughlin, Robert Hanson, Patrick Kuebler, Ignacio Dallo, Alberto Gobbi

**Affiliations:** ^1^Department of Biology, Franciscan University of Steubenville, Steubenville, OH 43952, USA; ^2^Atlas Medical Center, Irving TX 75062, USA; ^3^O.A.S.I. Bioresearch Foundation, Gobbi NPO, Milan, Italy

## Abstract

**Background:**

Intra-articular bone marrow concentrate (BMC) and aspirate (BMA) injections have been used with mixed results to treat osteoarthritis (OA). Given the various aspiration and concentration methods available for preparing bone marrow, more data are needed to identify the optimal bone marrow harvesting techniques to treat OA.

**Methods:**

This retrospective cohort study examined the effect of using low-volume BMAs harvested using the Marrow Cellution™ (MC) device on 160 patients (262 knees) suffering from pain due to knee OA, KL grades 2-4, that did not respond to conservative treatment. Changes in visual analog scores (VAS) for overall daily activity were examined over a six-month time frame in these patients (63.5 ± 0.97 years of age; 48.1% male). In addition, changes in the Western Ontario and McMaster Universities Arthritis Index (WOMAC) and Patient Global Impression of Change (PGIC scores) were examined over the same time frame in a smaller subset of patients (95 patients including 172 knees).

**Results:**

There was a statistically significant improvement in VAS scores for overall daily activity 6 months postprocedure in the study population, 7.29 ± 0.27 vs. 3.76 ± 0.34 (*p* < 0.0001), as well as statistically significant improvements in WOMAC scores, 49.3 ± 4.27 vs. 66.3 ± 4.08 (*p* < 0.0001). On the individual level, 71% of the cases displayed VAS improvements and 61% of the cases displayed WOMAC improvements that exceeded levels previous studies determined to be the minimal clinically important difference (MCID) for knee OA treatments. The improvements in WOMAC scores were also seen in both the WOMAC pain subscore, 52.2 ± 4.39 vs. 72.2 ± 4.36 (*p* < 0.0001) and the WOMAC function subscore, 51.6 ± 4.67 vs. 69.0 ± 4.36 (*p* < 0.0001). In addition, the PGIC scores measuring patient satisfaction improved from 4.03 ± 0.26 at 6 weeks postprocedure to 4.65 ± 0.28 at 6 months postprocedure (*p* < 0.0001).

**Conclusions:**

Knee OA patients treated with MC BMA intra-articular injections exhibited significant reductions in VAS pain scores and significant improvements in WOMAC scores that exceeded the minimal clinically important difference thresholds. In addition, reductions in VAS pain scores and improvements in WOMAC scores correlated with higher PGIC scores.

## 1. Background

Osteoarthritis (OA) is the most common form of arthritis and is one of the leading causes of disability in the USA, with the knee being the most commonly affected joint [1, 2]. More than ten percent of Americans over the age of 60 have some form of knee arthritis-related disability limiting their capacity to perform their activities of daily living [3]. OA is a degenerative disease that is associated with the breakdown of articular cartilage, a process that is accelerated by both age and injury [3, 4]. In addition to cartilage damage, OA is also correlated with pathological changes to periarticular tissues [5, 6].

Historically, osteoarthritis has been managed conservatively with a combination of physical therapy and oral pharmacologic intervention that focuses on reducing pain and inflammation. If conservative modalities fail, intra-articular injections of corticosteroids or hyaluronic acid can provide symptom relief; however, they do not tend to modify the underlying etiology of the disease [7, 8]. For patients with advanced OA, a total knee arthroplasty (TKA) can reduce pain and help restore function in the knee but it carries the risk of severe complications and can limit the patient's physical activities afterward [9, 10].

Given the issues and costs associated with TKA, other less invasive treatment options that can reduce the need for TKAs would be beneficial [11–13]. Toward this end, a variety of studies have examined the use of orthobiologics to treat knee OA [12, 14–17]. In particular, a number of studies have examined the ability of bone marrow concentrates (BMC) to treat OA [18–23]. Bone marrow contains mesenchymal stem cells (MSCs), platelets, and monocytes, all of which may contribute to improvements within the joint [24, 25]. While studies examining the use of BMCs in treating OA show a low risk of adverse events, recent meta-analyses have shown promising but often equivocal results in managing the symptoms of OA [26, 27]. The variability in outcomes in these studies is largely due to inadequacies in study design and variability in the techniques and systems used to produce BMCs.

Only a few studies have focused on using unprocessed low-volume bone marrow aspirates (BMA) to treat OA [28–30]. Given the ease of these BMA preparations over BMC preparations (no concentration step is needed) coupled with the fact that clinically relevant amounts of MSCs can be extracted in low volume aspirates, the use of low-volume BMAs could have significant clinical and practical advantages over the use of BMCs [31–35]. However, more studies are needed on the use of BMAs in the treatment of OA to address this knowledge gap.

This study examined the ability of low-volume BMA extracted using a novel technique, the Marrow Cellution™ (MC) system, to treat individuals with knee OA. This system allows for the extract of multiple low-volume draws (2 mls) from a single insertion site. The clinical records of 160 knee OA patients treated with BMA obtained using the novel MC aspiration technique were examined retrospectively in this study. The primary outcome measure was the reduction in pain as determined by the visual analog scale (VAS) six months after the procedure. Secondary outcome measures included the Patient Global Impression of Change (PGIC) and the Western Ontario and McMaster Universities Arthritis (WOMAC) index.

## 2. Materials and Methods

### 2.1. Patient Inclusion/Exclusion Criteria

Medical records for all patients who underwent bone marrow treatment for knee OA using the Marrow Cellution™ (MC) system at the Atlas Medical Center (Irving, TX) from March 2018 to Dec. 2019 were examined for possible inclusion in this retrospective analysis. The study received approval from the Franciscan University of Steubenville Institutional Review Board (protocol #2020-24, approved 11-17-2020). 202 knee OA patients who were treated using the MC system during this time period satisfied exclusion and inclusion criteria and filled out baseline surveys. Patients were included if they were over 18 years of age (the age range of patients actually enrolled was 37-89 years old), had knee pain and radiographically confirmed Kellgren-Lawrence grade II-IV knee OA (>80% were grade 2 and 3), and had failed at least six weeks of conservative therapy (activity modification, weight loss, brace, nonsteroidal inflammatory drugs, and corticosteroid injections). The study excluded those who had a major mechanical axis deviation of more than 50% into either compartment (varus or valgus) or 7°, an intra-articular injection into the affected knee within 6 weeks of the BMA injection, a body mass index of 40 or more or 18.5 or less, fever, active infection, clinically significant diabetes, cardiovascular, hepatic, or renal disease, use of antirheumatic medications (including methotrexate and other antimetabolites) within 4 days of BMA injection, malignancy or current chemo or radiation therapy, current drug or alcohol use disorder, a history of severe anemia or bleeding disorders, severe metabolic bone disease, and those who were pregnant or currently breast-feeding. No patients were excluded due to failure of aspiration based on either quality or quantity. In all cases, sufficient marrow was extracted to treat the patient.

Baseline surveys were obtained for 201 patients but 41 of these patients were lost during follow-up. As a result, a total of 160 patients representing 262 knees qualified for retrospective analysis of VAS scores. Baseline WOMAC and PGIC scores were only obtained for the most recent 110 patients. Six of these patients were lost during follow-up leaving 95 patients included in the WOMAC and PGIC analysis. The complete data set is available in Supplementary Table 1. Patient characteristics are described in Table 1.

### 2.2. Bone Marrow Aspiration and Injection

All BMA procedures were performed by two clinicians who each had over a year of experience with the technique before treating the study patients. Using local anesthesia in sterile conditions, the two clinicians followed the same aspiration technique as described previously [36]. In brief, after sterile skin preparation, 10 ml of lidocaine 2% was injected to anesthetize the periosteum and surrounding tissue. A bone marrow aspiration needle with side ports (Marrow Cellution kit, Ranfac Corp.) was then advanced to the cortex of the posterior iliac crest. The needle was inserted through the cortex using a mallet in a lateral and caudal direction. Once the needle passed through the cortex, the sharp stylet was exchanged for a blunt stylet. The needle was then manually advanced 4 cm into the medullary canal. The blunt stylet was replaced with a fenestrated aspiration cannula. The bone marrow was then aspirated following the manufacturer's recommended technique. This involved aspirating 2 ml of bone marrow and then slowly retracting the needle and aspirating another 2mls of bone marrow from a different region of the bone marrow within the same insertion site. This was repeated five times (five small 2 ml aspirates) for a total harvest of 10 ml of BMA from a single insertion site. This volume is small enough such that the draw is not diluted with peripheral blood [33, 34], but entails a sufficient volume to treat knee OA as has been done in a previous studies [30]. Previous studies have used between 6 and 20 mls of BMA or BMC [13, 37], although previous research using the MC system has found that roughly 10 mls of BMA using this technique is sufficient to achieve positive outcomes [30, 38]. To inject the BMA, the patient was placed supine and sterilizing the skin of the knee to be treated was sterilized. A sterile gel was applied and an ultrasound machine with a linear probe was used to visualize the knee joint superior recess. A 20-gauge, 1.5 inch needle was then advanced under ultrasound guidance into the knee joint. The BMA was then injected through the needle into the knee joint under ultrasound guidance. No particular structure such as the MCL or hub of the ACL was identified and targeted. Six of the 160 patients had minor swelling postprocedure, which resolved within five days. No significant side effects were observed at either the aspiration site or the injection site.

### 2.3. Patient-Reported Outcome Measures

Three different patient-reported outcome measures (PROMs) were used in this study and patients completed their PROM surveys on their own. The primary outcome measure used was the VAS score associated with overall daily activity. The WOMAC score and the Patient Global Impression of Change (PGIC) score were used as secondary outcome measures. VAS scores for overall daily activity at baseline and at 6 months postprocedure were available for all 160 patients (262 knees). WOMAC scores, at baseline and at 6 months postprocedure, were available for the final 95 patients enrolled in the study. As the WOMAC score evaluates bilateral knee function, the WOMAC scores are presented per patient rather than per knee. In addition, these 95 patients (172 knees) also provided PGIC scores 6 weeks after the procedure and 6 months after the procedure.

### 2.4. Statistics

The data was analyzed for normality using the Kolmogorov-Smirnov test and differences between baseline and 6 months postprocedure were analyzed using a paired *t*-test if the data was normally distributed (WOMAC and WOMAC subgroup data) and using a Wilcoxon matched-pairs signed rank test if the data was nonnormally distributed (VAS, PGIC, WOMAC pain subscale, and WOMAC function subscale data). Post hoc power analysis was performed (G∗Power 3.1 software) on all these comparisons (Supplementary Table 1). All group data are presented as mean ± 95%confidence interval (CI) with *p* < 0.01 being used to indicate a statistically significant difference between groups. Correlation analysis was done by calculating the Spearman's rank correlation coefficient. All data analyses were performed using Prism 9.0 (GraphPad Software).

## 3. Results

### 3.1. VAS Scores

**Figure 1:**
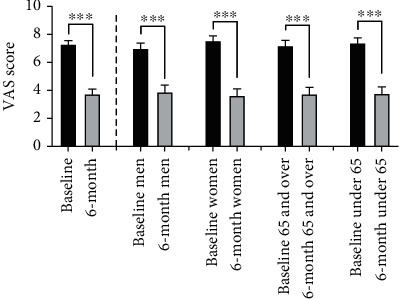
VAS scores at baseline and at six months postprocedure. The VAS scores were significantly improved at 6 months as compared to baseline in the total patient population (left columns). When broken into subgroups (men, women, patients < 65, and patients ≥ 65), all subgroups showed significant improvement as well (^∗∗∗^p ≤ 0.0001).

The VAS scores improved significantly in this population following MC treatment falling from an average of 7.29 ± 0.27 at baseline to 3.76 ± 0.34 at 6 months postprocedure (*p* < 0.0001) (Figure 1). Of the 262 knees analyzed, 187 (71%) displayed an improvement in the VAS score of 2 or more, a magnitude of improvement that is above what previous studies determined to be the MCID for knee OA treatments [39, 40].

When analyzed in subgroups, a significant improvement in VAS scores was seen across all groups including men (6.98 ± 0.40 to 3.91 ± 0.50, *p* < 0.0001, and *n* = 120 knees), women (7.54 ± 0.36 to 3.63 ± 0.47, *p* < 0.0001, and *n* = 142 knees), patients ≥ 65 years of age (7.20 ± 0.38 to 3.72 ± 0.49, *p* < 0.0001, and *n* = 133 knees), and patients < 65 years of age (7.38 ± 0.38 to 3.80 ± 0.48, *p* < 0.0001, and *n* = 129 knees) (Figure 1). The level of VAS improvement was greater in women than men, however the difference did not achieve statistical significance (−3.91 ± 0.51 vs. −3.08 ± 0.50, *p* = 0.026). No significant difference in VAS score improvement was seen between older vs younger patients (−3.47 ± 0.55 vs. −3.58 ± 0.47, *p* = 0.7595).

### 3.2. WOMAC

**Figure 2:**
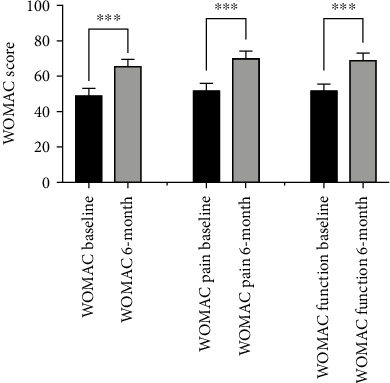
WOMAC scores at baseline and at six months postprocedure. The overall WOMAC scores were significantly improved at 6 months as compared to baseline. In addition, both the WOMAC pain and functional subscales were significantly improved at 6 months over baseline (^∗∗∗^p ≤ 0.0001).

In the subgroup of patients who had completed WOMAC scores, there was a significant improvement in the scores over baseline at 6 months postprocedure (Figure 2). Measured on a scale of 0-96, the overall WOMAC score improved from 49.3 ± 4.27 at baseline to 66.3 ± 4.08 at 6 months postprocedure (*p* < 0.0001) (Figure 2). Of the 95 patients analyzed, 58 (61%) displayed an improvement in the WOMAC score of 12.5 or more, a magnitude of improvement that is above what previous studies determined to be the MCID for knee OA treatments [41, 42]. In addition, both the pain and the functional subscales of the WOMAC showed significant improvements as well. The WOMAC pain subscale (adjusted to a 0-100 scale) improved from 52.2 ± 4.39 at baseline to 72.2 ± 4.36 at 6 months postprocedure (*p* < 0.0001). Likewise, the functional subscale (adjusted to a 0-100 scale) improved from 51.6 ± 4.67 at baseline to 69.0 ± 4.36 at 6 months postprocedure (*p* < 0.0001) (Figure 2).

When analyzed in subgroups, a significant improvement in overall WOMAC scores was seen across all groups including men (54.8 ± 6.20 to 69.1 ± 5.46, *p* = 0.0002, and *n* = 42), women (44.7 ± 5.25 to 64.2 ± 5.75, *p* < 0.0001, and *n* = 53), patients ≥ 65 years of age (49.1 ± 6.13 to 65.4 ± 5.56, *p* < 0.0001, and *n* = 49), and patients < 65 years of age (49.2 ± 5.54 to 67.4 ± 5.89, *p* < 0.0001, and *n* = 46). There were no significant difference seen in the improvement in WOMAC scores found in women vs. men (*p* = 0.2659) or older patients vs. younger patients (*p* = 0.6899).

### 3.3. PGIC

**Figure 3:**
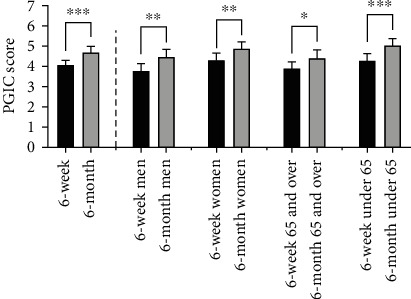
PGIC scores at 6 weeks and at six months postprocedure. The PGIC scores were significantly improved at 6 months postprocedure as compared to 6 weeks in the total patient population (left columns). When broken into subgroups (men, women, patients < 65, and patients ≥ 65), all subgroups showed significant improvement as well (^∗∗∗^p ≤ 0.0001; ^∗∗^p ≤ 0.001; and ^∗^p ≤ 0.01).

The self-reported patient satisfaction with the procedure as measured by PGIC scores improved from 6 weeks after the procedure (4.03 ± 0.26) to 6 months after the procedure (4.65 ± 0.28) (*p* < 0.0001) (Figure 3). In this seven-point scale, a score of 4 indicates stable disease while scores of 5-7 indicate improvement. When analyzed in subgroups, a significant improvement in PGIC scores was seen across all groups including men (3.73 ± 0.38 to 4.41 ± 0.43, *p* = 0.001, and *n* = 74 knees), women (4.25 ± 0.35 to 4.83 ± 0.36, *p* = 0.0019, and *n* = 98 knees), patients ≥ 65 years of age (3.85 ± 0.35 to 4.36 ± 0.42, *p* = 0.0118, and *n* = 92 knees), and patients < 65 years of age (4.24 ± 0.39 to 4.99 ± 0.36, *p* < 0.0001, and *n* = 80 knees) (Figure 3). Interestingly, women reported higher PGIC scores on average as compared to men while those under 65 reported higher PGIC scores on average as compared to patients 65 years and older. However, there was no significant difference in PGIC score improvement when comparing men vs. women (0.68 ± 0.38 vs. 0.576 ± 0.37, *p* = 0.2025) or those ≥65 vs. those <65 (0.505 ± 0.39 vs. 0.753 ± 0.358, *p* = 0.2539).

Overall, the PGIC scores were inversely correlated with VAS scores indicating that patients that reported less pain also reported greater satisfaction with the procedure (*r* = −0.5397; *p* < 0.0001). In addition, the PGIC scores at 6 months displayed moderate positive correlations with the 6-month WOMAC scores (*r* = 0.6240; *p* < 0.0001) and the 6-month WOMAC functional subscale scores (*r* = 0.6186; *p* < 0.0001), indicating that patients that reported greater functional abilities 6 months postprocedure also reported greater satisfaction with the procedure.

## 4. Discussion

In this retrospective study of 161 grade 2-4 OA patients treated with MC aspirate, no serious adverse events were observed, which agrees with previous studies using BMA and BMC to treat knee OA [21, 28, 30, 43]. In addition to demonstrating safety in this population, patients reported positive outcomes six months after treatment including significant reductions in pain and significant improvements in knee function. While the minimal clinically important difference (MCID) in VAS and WOMAC scores for knee OA treatments can vary depending on the treatment and severity of OA [44], a large percentage of the study population displayed improvements that exceeded previously reported MCIDs for knee OA interventions [39–42]. In the case of VAS changes, 71% of the cases reported improvements in VAS scores (2 or more points on the 10-point VAS scale) that exceeded the previously reported MCID [41, 42]. For the WOMAC, 61% of the patients reported improvements ( >12.5 points) that exceeded previously reported MCIDs [39, 40].

There was also a significant correlation between VAS pain and WOMAC pain and between the reduction in both pain scores and the PGIC patient satisfaction scores suggesting that the pain differences seen here are both meaningful to the patient and clinically relevant. In addition, the significant correlation between patient satisfaction and WOMAC functional score gains indicates these function gains were meaningful to the patients.

Multiple studies have shown positive improvement for patients with knee osteoarthritis using BMCs or BMAs [18, 19, 21, 28, 45, 46]. In fact, recent meta-analyses have shown promising results in managing the symptoms of OA, but many of the studies included in the meta-analyses involved small patient samples, short-term follow up, and the use of heterogeneous methods that are difficult to compare directly [26, 27]. While the present study is likewise limited in its short-term follow-up, it had a large study population, and it involved a standardized aspiration technique. The improved patient outcomes seen here are in general agreement with previous work with BMC injections. In one previous study, Kim et al. reported improvement in patient outcomes after an autologous BMC injection, as measured with the VAS, KOOS, and Lysholm Knee Questionnaire [46]. Likewise, Centeno et al. reported modest improvements in pain (NPS) and functional (IKDC) scores following BMC intra-articular injections [43]. Given that the results of these studies are in general agreement with the data presented here, this suggests that the use of the low-volume BMA to treat OA seen here compares favorably to the use of different BMC preparations.

The ability to treat knee OA successfully using a low-volume BMA has significant benefits over the use of BMCs. These benefits include the use of only one insertion site for harvesting, a lower volume of bone marrow extraction, and the avoidance of a centrifugation step. Using this technique, aspirates do not have to be processed following extraction making them simpler to administer and less subject to the variability seen in the BMC outputs of centrifugation systems. Given that much work has shown that small volume draws have significantly higher MSC and CFU-f counts per ml than large volume aspirates, which tend to draw significant amounts of peripheral blood, the need for a centrifugation step to enhance bone marrow quality is not obvious [32–34]. Unfortunately, there are only a limited number of previous studies that have examined the use of the low-volume BMA described here to treat knee OA. While these studies found results similar to the results seen here, they all involved relatively small sample sizes. One recent study reported a significant increase in all five subscales of the KOOS 12 weeks postprocedure as well as a decrease in VAS scores but only had 17 knee OA patients [28]. Another related study only involved ten patients but found more than a 50% reduction in pain 12 weeks postprocedure [29]. Finally, a study following 11 patients (15 knees) with mild knee OA found that low-volume BMA injections significant improved KOOS, JR scores and Numerical Rating Scale (NRS) pain scores out to 12 months postinjection [30].

A common limitation of all of these low-volume BMA studies, a limitation shared with the present study and the majority of BMC OA studies in the literature, is the lack of a proper control group. Toward this end, a large-scale randomized placebo-controlled study in which patients are stratified based on KL grade is warranted to determine if the benefits for knee OA described here using low-volume BMA injections are reproducible in a controlled setting. While the current study was not stratified, the vast majority of the patients in this study had KL grades 2-3 OA suggesting that this treatment is beneficial for this population. Future studies should also establish a standardized rehabilitation program postprocedure and should follow patients for up to two years postprocedure. In addition, studies should measure the yields of MSCs in the low-volume BMA to determine if levels of MSCs correlate with patient outcomes as have been seen in previous studies [47–49]. Finally, the use of radiographic imaging may help understand if there are any structural changes that occur in the osteoarthritic joint following a BMA intra-articular injection and whether these changes correlate with positive patient reported outcomes.

## 5. Conclusions

The intra-articular injection of a low-volume BMA harvested using the MC technique was associated with positive patient outcomes six months postprocedure for patients with KL Grade 2-4 OA who had failed conservative treatments. The majority of the patients in this study were grades 2-3, indicating that this treatment is beneficial in the short-term for this population. The study included 161 patients (263 knees) and demonstrated statistically significant improvements in pain and function using this low-volume BMA treatment. In addition, a large percentage of the patients reported improvements in VAS (71%) and WOMAC (61%) scores that exceeded the MCID levels reported in previous knee OA studies. This suggests that these BMA treatments are a viable option for patients with grades 2-3 OA. Finally, this novel BMA technique is simpler and less invasive to perform than BMC techniques that require (1) an extra centrifugation step and (2) a higher volume aspiration and/or multiple site aspirations. Despite this promising data, clinical studies that incorporate longer term follow-ups along with BMA characterization are needed to better understand the therapeutic potential of these low-volume BMAs for the treatment of OA.

## Figures and Tables

**Table 1:** Patient demographics for the 160 total patients enrolled in the VAS portion of the study. The demographics are also listed for the subgroup, 95 total patients, of this population for which WOMAC and PGIC scores were obtained.

	Number of patients	Average age (± SEM)	Percent male
*Total VAS*	**160**	63.2 ± 0.97	**48.1%**
Males	77	62.5 ± 1.44	—
Females	83	64.4 ± 1.31	—
Patients < 65 years old	82	53.3 ± 0.83	52.4%
Patients ≥ 65 years old	78	73.7 ± 0.77	43.6%
*Total WOMAC and PGIC*	**95**	64.3 ± 1.25	**44.2%**
Males	42	64.0 ± 1.85	—
Females	53	64.6 ± 1.71	—
Patients < 65 years old	46	54.2 ± 1.11	50.0%
Patients > = 65 years old	49	73.8 ± 0.99	38.8%

## Data Availability

The data is available in spreadsheet fashion by contacting the corresponding author and as a supplementary table.

## Supplementary Materials

Supplementary 1 Supplementary Table 1. Post hoc effect sizes and power for the various comparisons analyzed in Figures 1–3.

Supplementary 2 Supplementary Table 2. Raw data for all patients included in the study. The data set includes age, sex, as well as WOMAC scores, VAS scores, and PGIC scores at baseline and 6-month follow-up for all patients.
