# Supplementary material for: Short-Term Efficacy of Using a Novel Low-Volume Bone Marrow Aspiration Technique to Treat Knee Osteoarthritis: A Retrospective Cohort Study
Source: Stem Cells Int. 2022 Nov 15;2022:5394441. doi: 10.1155/2022/5394441 (PMC9682226; doi:10.1155/2022/5394441)
Supplement: Supplementary 1 — Supplementary Table 1. Post hoc effect sizes and power for the various comparisons analyzed in Figures 1–3. [file 5394441.f1.docx]

**Supplementary Table 1**: Post-hoc effect sizes and power for the various comparisons analyzed in figures 1, 2, and 3.

| **Test** | **Comparison** | **Effect Size** | **Power** |
| --- | --- | --- | --- |
| VAS | All patients; baseline vs. 6 months | 1.13 | **> 0.9999** |
| VAS | Men; baseline vs. 6 months | 1.11 | **> 0.9999** |
| VAS | Women: baseline vs. 6 months | 1.27 | **> 0.9999** |
| VAS | Patients < 65: baseline vs. 6 months | 1.29 | **> 0.9999** |
| VAS | Patients >= 65: baseline vs. 6 months | 1.09 | **> 0.9999** |
| VAS | Men vs Women Improvements | 0.280 | 0.6124 |
| VAS | Old vs Young Improvements | 0.266 | 0.5543 |
| WOMAC | All patients; baseline vs. 6 months | 0.8731 | **> 0.9999** |
| WOMAC Pain | All patients; baseline vs. 6 months | 0.8813 | **> 0.9999** |
| WOMAC Function | All patients; baseline vs. 6 months | 0.8241 | **> 0.9999** |
| WOMAC | Men; baseline vs. 6 months | 0.5622 | **0.9449** |
| WOMAC | Women: baseline vs. 6 months | 0.7286 | **0.9994** |
| WOMAC | Patients < 65: baseline vs. 6 months | 0.7237 | **0.9978** |
| WOMAC | Patients >= 65: baseline vs. 6 months | 0.6024 | **0.9850** |
| WOMAC | Men vs Women Improvements | 0.2339 | 0.2017 |
| WOMAC | Old vs Young Improvements | 0.0837 | 0.0689 |
| PGIC | All patients; 6 weeks vs. 6 months | 0.3112 | **0.9787** |
| PGIC | Men; 6 weeks vs. 6 months | 0.4114 | **0.9297** |
| PGIC | Women: 6 weeks vs. 6 months | 0.3099 | **0.8466** |
| PGIC | Patients < 65: 6 weeks vs. 6 months | 0.4657 | **0.9814** |
| PGIC | Patients >= 65: 6 weeks vs. 6 months | 0.2671 | **0.7019** |
| PGIC | Men vs Women Improvements | 0.0613 | 0.0675 |
| PGIC | Old vs Young Improvements | 0.1415 | 0.1478 |
