# Supplementary material for: Short-Term Efficacy of Using a Novel Low-Volume Bone Marrow Aspiration Technique to Treat Knee Osteoarthritis: A Retrospective Cohort Study
Source: Stem Cells Int. 2022 Nov 15;2022:5394441. doi: 10.1155/2022/5394441 (PMC9682226; doi:10.1155/2022/5394441)
Supplement: Supplementary 2 — Supplementary Table 2. Raw data for all patients included in the study. The data set includes age, sex, as well as WOMAC scores, VAS scores, and PGIC scores at baseline and 6-month follow-up for all patients. [file 5394441.f2.pdf]

Supplementary Table 1

Baseline							Follow Up						
Patient Number	Age	Sex	Knee (L) (R)	Womac	VAS	PGIC (6 wks)	Patient Number	Age	Sex	Knee (L) (R)	Womac	VAS	PGIC
1	65	F	L	17.4	10	5	1	65	F	L	92.0	0	7
1	65	F	R	17.4	10	6	1	65	F	R	92.0	0	7
2	58	M	L	62.1	2	6	2	58	M	L	90.9	2	6
2	58	M	R	62.1	5	6	2	58	M	R	90.9	2	7
3	63	M	R	74.2	5	2	3	63	M	R	74.2	5	2
4	75	M	L	77.0	8	1	4	75	M	L	53.0	7	1
4	75	M	R	77.0	4	2	4	75	M	R	53.0	1	2
5	38	M	R	78.7	7	5	5	38	M	R	95.4	2	4
6	65	M	L	23.4	9	6	6	65	M	L	45.4	5	5
6	65	M	R	23.4	9	5	6	65	M	R	45.4	7	2
7	67	F	R	78.0	8	7	7	67	F	R	100.0	0	7
8	64	F	L	58.3	4	5	8	64	F	L	90.9	2	5
8	64	F	R	58.3	6	5	8	64	F	R	90.9	2	5
9	71	M	L	60.6	8	3	9	71	M	L	59.8	3	4
9	71	M	R	60.6	8	5	9	71	M	R	59.8	3	4
10	60	M	L	49.2	8	5	10	60	M	L	56.8	4	5
10	60	M	R	49.2	8	6	10	60	M	R	56.8	4	5
11	55	F	L	48.4	6	5	11	55	F	L	60.6	5	5
11	55	F	R	48.4	6	5	11	55	F	R	60.6	5	5
12	72	F	L	46.2	7	3	12	72	F	L	91.6	2	5
12	72	F	R	46.2	7	3	12	72	F	R	91.6	2	5
13	60	F	L	54.5	5	7	13	60	F	L	60.6	6	4
13	60	F	R	54.5	8	5	13	60	F	R	60.6	6	4
14	61	M	L	31.8	10	3	14	61	M	L	43.2	5	5
14	61	M	R	31.8	10	3	14	61	M	R	43.2	5	5
15	88	F	L	49.2	4	1	15	88	F	L	33.3	1	1
15	88	F	R	49.2	10	1	15	88	F	R	33.3	10	1
16	68	F	L	46.2	2	6	16	68	F	L	78.7	1	7
16	68	F	R	46.2	4	6	16	68	F	R	78.7	1	6
17	56	F	L	26.5	6	6	17	56	F	L	84.1	0	6
17	56	F	R	26.5	10	5	17	56	F	R	84.1	0	6
18	46	M	L	72.7	6	3	18	46	M	L	98.4	4	7
18	46	M	R	72.7	5	3	18	46	M	R	98.4	4	7
19	66	M	L	27.2	6	6	19	66	M	L	74.2	8	6
19	66	M	R	27.2	9	5	19	66	M	R	74.2	7	5
20	39	M	L	47.0	7	5	20	39	M	L	85.6	1	7
20	39	M	R	47.0	7	6	20	39	M	R	85.6	1	7
21	52	M	L	71.9	9	3	21	52	M	L	61.4	8	5
21	52	M	R	71.9	9	3	21	52	M	R	61.4	7	4
22	46	F	L	41.6	9	1	22	46	F	L	82.5	4	5
22	46	F	R	41.6	9	3	22	46	F	R	82.5	0	5
23	63	M	L	58.3	3	4	23	63	M	L	72.7	1	7
23	63	M	R	58.3	5	5	23	63	M	R	72.7	2	6
24	59	M	L	21.1	10	3	24	59	M	L	46.2	6	4
24	59	M	R	21.1	10	2	24	59	M	R	46.2	10	4
25	68	F	L	9.0	10	4	25	68	F	L	2.0	10	1
25	68	F	R	9.0	8	4	25	68	F	R	2.0	9	2
26	76	F	L	42.4	9	7	26	76	F	L	92.4	0	6
26	76	F	R	42.4	9	7	26	76	F	R	92.4	0	7
27	82	F	L	49.0	9	1	27	82	F	L	63.6	7	2
27	82	F	R	49.0	7	1	27	82	F	R	63.6	7	2
28	51	F	L	56.8	8	5	28	51	F	L	70.4	4	6
28	51	F	R	56.8	5	5	28	51	F	R	70.4	4	6
29	78	M	L	50.0	8	5	29	78	M	L	65.9	0	5

29	78	M	R	50.0	8	4
30	69	F	L	49.2	7	5
30	69	F	R	49.2	7	5
31	83	M	L	89.4	7	5
31	83	M	R	89.4	7	6
32	75	F	L	30.3	10	1
32	75	F	R	30.3	3	1
33	44	F	L	59.0	4	6
33	44	F	R	59.0	4	6
34	67	F	L	80.3	7	2
34	67	F	R	80.3	7	3
35	43	M	L	30.3	9	5
36	46	F	L	64.3	9	4
36	46	F	R	64.3	9	4
37	58	F	L	19.6	5	3
37	58	F	R	19.6	5	3
38	82	M	R	89.3	6	3
39	75	M	R	78.7	5	1
40	71	F	L	44.6	10	5
40	71	F	R	44.6	8	5
41	68	F	L	37.8	6	5
41	68	F	R	37.8	8	3
42	50	M	L	41.6	7	7
42	50	M	R	41.6	7	7
43	83	F	L	50.0	8	4
43	83	F	R	50.0	8	2
44	71	M	L	53.7	8	3
44	71	M	R	53.7	8	3
45	59	F	L	57.6	7	6
45	59	F	R	57.6	7	6
46	61	F	L	58.3	6	7
46	61	F	R	58.3	6	7
47	56	F	L	34.1	3	6
47	56	F	R	34.1	8	2
48	84	F	L	56.8	4	4
48	84	F	R	56.8	4	5
49	83	F	L	11.3	10	7
49	83	F	R	11.3	10	7
50	66	M	L	65.1	5	4
50	66	M	R	65.1	5	4
51	55	M	R	53.0	9	7
52	68	F	L	46.9	6	3
52	68	F	R	46.9	3	3
53	52	F	L	8.3	10	2
53	52	F	R	8.3	10	5
54	50	M	L	70.4	6	1
54	50	M	R	70.4	2	1
55	65	F	L	50.7	7	6
56	40	F	L	32.5	10	3
57	54	M	L	35.6	10	2
57	54	M	R	35.6	10	2
58	66	M	L	58.3	8	1
59	68	F	L	74.2	3	6
60	63	F	L	27.2	8	7
60	63	F	R	27.2	10	6
61	46	F	L	73.4	8	2
61	46	F	R	73.4	8	2

29	78	M	R	65.9	1	5
30	69	F	L	70.4	8	6
30	69	F	R	70.4	7	5
31	83	M	L	70.4	8	4
31	83	M	R	70.4	3	6
32	75	F	L	55.3	7	2
32	75	F	R	55.3	5	2
33	44	F	L	99.2	0	6
33	44	F	R	99.2	0	6
34	67	F	L	75.0	3	5
34	67	F	R	75.0	2	5
35	43	M	L	92.4	0	6
36	46	F	L	46.2	5	4
36	46	F	R	46.2	7	4
37	58	F	L	61.3	4	2
37	58	F	R	61.3	5	2
38	82	M	R	90.9	5	1
39	75	M	R	87.9	2	4
40	71	F	L	71.9	10	2
40	71	F	R	71.9	0	7
41	68	F	L	53.7	3	5
41	68	F	R	53.7	6	3
42	50	M	L	97.0	0	7
42	50	M	R	97.0	0	7
43	83	F	L	68.1	0	3
43	83	F	R	68.1	4	5
44	71	M	L	35.6	9	1
44	71	M	R	35.6	9	1
45	59	F	L	98.5	0	7
45	59	F	R	98.5	0	7
46	61	F	L	72.0	0	7
46	61	F	R	72.0	2	6
47	56	F	L	63.6	3	6
47	56	F	R	63.6	7	5
48	84	F	L	67.4	1	1
48	84	F	R	67.4	4	2
49	83	F	L	62.1	2	7
49	83	F	R	62.1	3	7
50	66	M	L	56.1	5	5
50	66	M	R	56.1	0	6
51	55	M	R	90.2	6	7
52	68	F	L	82.5	0	6
52	68	F	R	82.5	0	7
53	52	F	L	37.1	8	4
53	52	F	R	37.1	10	2
54	50	M	L	76.5	4	1
54	50	M	R	76.5	1	1
55	65	F	L	92.4	0	7
56	40	F	L	26.5	5	2
57	54	M	L	39.4	6	4
57	54	M	R	39.4	6	3
58	66	M	L	92.6	0	7
59	68	F	L	51.5	2	2
60	63	F	L	42.4	5	4
60	63	F	R	42.4	5	3
61	46	F	L	74.2	7	5
61	46	F	R	74.2	7	6

62	65	F	L	35.6	10	7	62	65	F	L	91.7	0	7
62	65	F	R	35.6	10	5	62	65	F	R	91.7	0	6
63	59	M	L	38.6	7	6	63	59	M	L	68.2	1	5
63	59	M	R	38.6	3	5	63	59	M	R	68.2	0	6
64	89	F	L	43.1	8	2	64	89	F	L	72.7	2	6
64	89	F	R	43.1	8	4	64	89	F	R	72.7	2	6
65	58	M	L	43.9	7	4	65	58	M	L	87.8	3	4
65	58	M	R	43.9	7	6	65	58	M	R	87.8	0	7
66	55	M	L	57.5	8	1	66	55	M	L	77.2	5	5
66	55	M	R	57.5	8	1	66	55	M	R	77.2	5	5
67	71	F	L	46.9	6	5	67	71	F	L	73.4	2	6
67	71	F	R	46.9	8	5	67	71	F	R	73.4	3	6
68	84	F	L	9.0	10	4	68	84	F	L	38.6	5	7
68	84	F	R	9.0	10	3	68	84	F	R	38.6	7	5
69	61	F	L	50.7	8	3	69	61	F	L	62.1	3	5
69	61	F	R	50.7	8	3	69	61	F	R	62.1	3	4
70	49	F	R	23.4	10	3	70	49	F	R	85.6	6	5
71	83	M	L	70.4	3	3	71	83	M	L	65.1	2	5
72	79	M	L	64.4	7	2	72	79	M	L	40.9	6	2
72	79	M	R	64.4	7	2	72	79	M	R	40.9	6	2
73	83	M	L	46.0	10	4	73	83	M	L	53.0	5	5
73	83	M	R	46.0	10	5	73	83	M	R	53.0	7	3
74	64	M	R	61.3	8	5	74	64	M	R	80.3	2	6
75	53	F	R	71.9	8	4	75	53	F	R	61.4	0	6
76	77	F	L	78.8	5	2	76	77	F	L	48.4	8	4
76	77	F	R	78.8	5	2	76	77	F	R	48.4	5	4
77	69	F	L	50.0	8	5	77	69	F	L	65.1	4	6
77	69	F	R	50.0	8	5	77	69	F	R	65.1	4	6
78	41	F	R	39.4	10	5	78	41	F	R	50.0	5	5
79	69	F	L	29.5	9	6	79	69	F	L	55.0	3	5
79	69	F	R	29.5	9	6	79	69	F	R	55.0	3	5
80	61	M	L	93.1	6	6	80	61	M	L	84.0	3	6
81	61	M	L	62.8	5	3	81	61	M	L	72.0	4	2
81	61	M	R	62.8	5	3	81	61	M	R	72.0	4	2
82	66	F	L	46.2	7	3	82	66	F	L	59.0	4	5
82	66	F	R	46.2	7	3	82	66	F	R	59.0	4	3
83	72	M	L	86.3	1	4	83	72	M	L	88.6	0	5
83	72	M	R	86.3	2	4	83	72	M	R	88.6	1	6
84	71	M	L	25.0	6	4	84	71	M	L	70.5	1	4
84	71	M	R	25.0	6	3	84	71	M	R	70.5	1	4
85	74	M	L	33.3	7	3	85	74	M	L	80.3	3	6
85	74	M	R	33.3	7	3	85	74	M	R	80.3	3	6
86	72	M	L	80.3	3	2	86	72	M	L	62.1	5	1
86	72	M	R	80.3	2	3	86	72	M	R	62.1	6	1
87	64	M	R	53.0	7	5	87	64	M	R	68.2	5	5
88	72	F	L	53.7	8	5	88	72	F	L	82.6	2	6
88	72	F	R	53.7	8	6	88	72	F	R	82.6	1	7
89	73	F	L	76.5	10	3	89	73	F	L	86.3	2	4
89	73	F	R	76.5	10	4	89	73	F	R	86.3	2	4
90	44	F	L	44.7	9	4	90	44	F	L	86.4	0	7
90	44	F	R	44.7	9	5	90	44	F	R	86.4	2	7
91	59	M	L	52.2	8	2	91	59	M	L	70.4	8	3
91	59	M	R	52.2	8	3	91	59	M	R	70.4	5	4
92	59	F	L	40.2	10	3	92	59	F	L	55.3	6	6
92	59	F	R	40.2	10	3	92	59	F	R	55.3	8	4
93	66	M	L	24.2	3	4	93	66	M	L	24.2	0	4
93	66	M	R	24.2	6	2	93	66	M	R	24.2	2	5

94	84	M	L	33.3	8	3	94	84	M	L	64.4	3	3	
94	84	M	R	33.3	8	2	94	84	M	R	64.4	5	2	
95	59	F	L	55.3	9	6	95	59	F	L	43.9	2	6	
95	59	F	R	55.3	9	5	95	59	F	R	43.9	2	6	
96	78	F	L	57.6	6	3	96	78	F	L	63.6	0	6	
96	78	F	R	57.6	6	1	96	78	F	R	63.6	3	5	
97	78	M	L		8		97	78	M	L		6		
97	78	M	R		8		97	78	M	R		6		
98	73	F	L		10		98	73	F	L		5		
98	73	F	R		10		98	73	F	R		5		
99	51	M	L		1		99	51	M	L		0		
99	51	M	R		8		99	51	M	R		7		
100	71	M	L		5		100	71	M	L		2		
101	65	M	L		7		101	65	M	L		7		
101	65	M	R		9		101	65	M	R		8		
102	79	M	L		7		102	79	M	L		0		
103	74	M	L		8		103	74	M	L		7		
104	78	F	L		6		104	78	F	L		9		
105	59	F	L		8		105	59	F	L		8		
105	59	F	R		9		105	59	F	R		7		
106	67	F	L		9		106	67	F	L		5		
107	37	M	R		7		107	37	M	R		1		
108	49	M	L		2		108	49	M	L		0		
109	62	F	L		8		109	62	F	L		2		
109	62	F	R		8		109	62	F	R		3		
110	50	M	L		10		110	50	M	L		5		
111	36	M	L		10		111	36	M	L		5		
112	69	F	R		7		112	69	F	R		3		
113	49	F	L		5		113	49	F	L		4		
113	49	F	R		5		113	49	F	R		4		
114	59	F	L		8		114	59	F	L		0		
114	59	F	R		6		114	59	F	R		0		
115	85	F	L		3		115	85	F	L		1		
115	85	F	R		8		115	85	F	R		4		
116	61	F	L		2		116	61	F	L		1		
117	57	F	R		7		117	57	F	R		2		
118	41	M	R		8		118	41	M	R		1		
119	50	M	R		10		119	50	M	R		7		
120	47	F	R		7		120	47	F	R		3		
121	57	M	R		8		121	57	M	R		5		
122	18	M	R		7		122	18	M	R		1		tudy due to young age--outlier
123	65	M	R		4		123	65	M	R		0		
124	62	F	R		9		124	62	F	R		2		
125	58	M	L		5		125	58	M	L		4		
126	68	F	L		8		126	68	F	L		1		
127	83	M	R		6		127	83	M	R		6		
128	60	M	R		9		128	60	M	R		3		
129	69	F	L		3		129	69	F	L		1		
130	54	M	L		8		130	54	M	L		8		
130	54	M	R		8		130	54	M	R		5		
131	58	M	L		5		131	58	M	L		0		
132	57	F	L		10		132	57	F	L		1		
133	54	M	L		8		133	54	M	L		9		
134	57	F	L		8		134	57	F	L		8		
134	57	F	R		8		134	57	F	R		8		
135	70	F	R		9		135	70	F	R		1		
136	54	F	R		6		136	54	F	R		2		

137	71	M	L	4
138	69	M	L	8
138	69	M	R	8
139	65	M	L	8
139	65	M	R	8
140	72	F	L	8
140	72	F	R	8
141	87	M	R	8
142	63	M	L	5
142	63	M	R	5
143	61	M	L	8
143	61	M	R	8
144	47	F	R	10
145	48	M	R	9
146	64	F	L	8
147	38	F	R	4
148	51	M	L	10
148	51	M	R	10
149	83	M	L	8
149	83	M	R	8
150	84	F	L	10
150	84	F	R	10
151	77	F	L	5
151	77	F	R	5
152	78	M	R	10
153	62	F	L	10
153	62	F	R	10
154	76	F	L	10
154	76	F	R	10
155	49	M	L	8
156	67	M	R	7
157	72	F	L	5
158	52	M	L	6
159	71	F	L	6
159	71	F	R	10
160	53	F	R	10
161	46	M	L	9
161	46	M	R	9

137	71	M	L	3
138	69	M	L	0
138	69	M	R	5
139	65	M	L	8
139	65	M	R	10
140	72	F	L	7
140	72	F	R	7
141	87	M	R	6
142	63	M	L	8
142	63	M	R	7
143	61	M	L	8
143	61	M	R	5
144	47	F	R	9
145	48	M	R	3
146	64	F	L	4
147	38	F	R	0
148	51	M	L	4
148	51	M	R	3
149	83	M	L	3
149	83	M	R	1
150	84	F	L	6
150	84	F	R	4
151	77	F	L	6
151	77	F	R	2
152	78	M	R	2
153	62	F	L	7
153	62	F	R	8
154	76	F	L	6
154	76	F	R	2
155	49	M	L	2
156	67	M	R	6
157	72	F	L	5
158	52	M	L	3
159	71	F	L	6
159	71	F	R	1
160	53	F	R	8
161	46	M	L	0
161	46	M	R	0
